# Triparental ageing in a laboratory population of an insect with maternal care

**DOI:** 10.1093/beheco/arac078

**Published:** 2022-08-24

**Authors:** Hilary Cope, Edward R Ivimey-Cook, Jacob Moorad

**Affiliations:** Institute of Evolutionary Biology, School of Biological Sciences, University of Edinburgh, Edinburgh, UK; Institute of Evolutionary Biology, School of Biological Sciences, University of Edinburgh, Edinburgh, UK; Institute of Evolutionary Biology, School of Biological Sciences, University of Edinburgh, Edinburgh, UK

**Keywords:** maternal, offspring, paternal, senescence, interaction

## Abstract

Parental age at reproduction influences offspring size and survival by affecting prenatal and postnatal conditions in a wide variety of species, including humans. However, most investigations into this manifestation of ageing focus upon maternal age effects; the effects of paternal age and interactions between maternal and paternal age are often neglected. Furthermore, even when maternal age effects are studied, pre- and post-natal effects are often confounded. Using a cross-fostered experimental design, we investigated the joint effects of pre-natal paternal and maternal and post-natal maternal ages on five traits related to offspring outcomes in a laboratory population of a species of burying beetle, *Nicrophorus vespilloides*. We found a significant positive effect of the age of the egg producer on larval survival to dispersal. We found more statistical evidence for interaction effects, which acted on larval survival and egg length. Both interaction effects were negative and involved the age of the egg-producer, indicating that age-related pre-natal maternal improvements were mitigated by increasing age in fathers and foster mothers. These results agree with an early study that found little evidence for maternal senescence, but it emphasizes that parental age interactions may be an important contributor to ageing patterns. We discuss how the peculiar life history of this species may promote selection to resist the evolution of parental age effects, and how this might have influenced our ability to detect senescence.

## Teaser

Recent attention within the field of ageing has focused on the relationship between the age of parents and the quality of offspring, but the picture that is emerging is that the presence of this aspect of ageing is highly variable. We describe a laboratory experiment that independently measured effects of maternal and paternal age effects on the fitness traits of larvae and female foster mothers in a species of insect that feeds its young during their development as larvae.

## INTRODUCTION

Senescence is broadly defined as the progressive loss of function due to the accumulation of damage with age and is typically associated with declining fertility and survival, known respectively as reproductive and actuarial senescence (Finch 1990; Monaghan et al. 2008; Jones et al. 2014). Many studies have reported wide taxonomic variation in patterns of both types of senescence across the tree of life (Promislow 1991; Gaillard et al. 1994; Jones et al. 2008, 2014; Nussey et al. 2008; Lemaitre and Gaillard 2017). These perspectives tend to consider only the relationship between age and outcome within individuals. However, recent attention has begun to focus upon how the age of one individual affects the phenotype of another with a specific emphasis placed upon the effects of parental age upon offspring traits (Moorad and Nussey 2016; Tidiere et al. 2018; Ivimey-Cook and Moorad 2020; Monaghan et al. 2020; Vuarin et al. 2021).

Most of this research focuses upon maternal ageing, or the tendency for offspring performance to change as maternal age increases. Perhaps the best-known manifestations of maternal senescence, or age-related performance declines, take the form of negative associations between maternal age and two offspring outcomes: adult lifespan and juvenile survival. The first is known as the “Lansing effect” (Lansing 1947; Comfort 1953; Monaghan et al. 2020); while anecdotal evidence appears to suggest that this is a common pattern across species [see Table 1 from Monaghan et al. (2020)], no formal review has yet assessed its prevalence. Maternal senescence expressed as age-related declines in juvenile survival, on the other hand, does appear to occur more frequently than not in adequately investigated animal species groups, with considerable variation among taxonomic groups (Ivimey-Cook and Moorad 2020). Evolutionary genetic theory does offer some explanation for variation in maternal age effects on juvenile survival (Moorad and Nussey 2016), but it has not yet been generalized to explain formally the evolution of the Lansing Effect or other types of maternal senescence.

Paternal age effects are much less well-studied than maternal age effects (Lemaitre and Gaillard 2017), likely owing to the absence of post-zygotic paternal investment in most taxa that may reduce the opportunity for fathers to influence offspring (Kokko and Jennions 2008). Nonetheless, paternal age effects have been shown to exist in both human (Kong et al. 2012) and animal populations (Priest et al. 2002; Preston et al. 2015; Fay et al. 2016; Vuarin et al. 2019, 2021; Aich et al. 2020, 2021), with the majority of studies reporting deleterious effects of ageing. Our theoretical understanding of the evolution of paternal senescence lags behind that of maternal senescence, and no comparable evolutionary models have yet been proposed.

A fuller understanding of parental age effects comes from simultaneously investigating the joint effects of both paternal and maternal age (Laaksonen et al. 2002; Priest et al. 2002; Auld and Charmantier 2011; Torres et al. 2011; Ducatez et al. 2012; Auld et al. 2013; Bouwhuis et al. 2015; Brommer et al. 2015; Preston et al. 2015; Fay et al. 2016; Whelan et al. 2016; Vuarin et al. 2021). When both parents’ ages are varied at least semi-independently, then maternal-by-paternal age interactions can be estimated, and these can tell us how ageing in one parent can dampen (buffer) or amplify the effect of age in the other (Auld, 2011, 2013; Ducatez 2012; Bouwhuis 2015; Whelan 2016). The joint effects of biparental ageing are occasionally characterized in terms of the difference between parental ages, or PADs (parental age differences) (Fieder and Huber 2007; Helle et al. 2008; Tidiere et al. 2018). The effects of PADs are related to the main and interaction effects of biparental age as $\beta_{z,PAD}=\beta_{z,M}-\beta_{z,F}-\beta_{z,M\times F}$_._, where *z* is offspring performance and β indicates the effect upon *z* caused by differences in male (*M*) or female (*F*) age. The effects of PADs are clearly inflated relative to the differences in parental and maternal age effects when there is a negative interaction between the two, as we might expect when there is some sort of compensation for the effects of age by one parent by the other. Conversely, PAD effects are suppressed by positive interactions, as we might expect if old age in one parent makes the offspring’s more sensitive to changes in the age of the other parent. Despite several studies finding no significant interaction effects associated with biparental age (Auld and Charmantier 2011; Auld et al. 2013; Bouwhuis et al. 2015; Tidiere et al. 2016), several others have highlighted the importance of studying maternal-by-paternal age interactions. For instance, male and female ages interact to advance lay date in a population of Canada jays, *Perisoreus canadensis* (Whelan et al. 2016), with older males buffering the detrimental effect of reduced female experience. In a Lepidopteran species, *Pieris brassicae*, male and female age at laying interacted to increase delays in offspring development (Ducatez et al. 2012).

Furthermore, when mothers provide post-natal care, then age may have different effects on pre- and post-natal maternal care pathways. In systems that permit cross-fostering, experiments can be designed such that the ages of the egg and sperm producers can be decoupled from the age of parents that provide post-natal care (foster parents), and these pathways can be assessed for differences in ageing rates. With maternal cross-fostering, studies of triparental age effects can provide estimates of three main effects of parental ageing and three relevant two-way parental age interactions effects. Assessing two of these interactions (father age—by—egg-producer age and father age—by—foster mother age) may allow us to understand better where parental age interactions arise in natural systems and how the age of one parent may buffer against the deleterious effects of age in the other. An egg-producer age—by—foster mother age interaction can also be estimated; this interaction won’t contribute to PADs, but it could provide insights into how one aspect of maternal care can buffer against the effects of ageing in the other.

In this study, we investigate the joint effects of triparental ageing on the performance of *Nicrophorus vespilloides,* a species of burying beetle that exhibits maternal care. Burying beetles are useful laboratory systems for studying parental effects because the larvae are highly amenable to cross-fostering. For this reason, it is straightforward to disentangle the effects of egg-producers (egg quality and carcass preparation) from those of foster mothers (carcass care and larval feeding) (Lock et al. 2007; Ivimey-Cook and Moorad 2018). Previous work involving *N. vespilloides* has found mixed evidence of maternal age effects: some studies have found that increased maternal age at reproduction had a detrimental effect on a number of larval traits (Ward et al. 2009; Cotter et al. 2011), however a more recent paper that disentangled pre- and post-natal contributions found no linear effects of age on any of the measured offspring traits [see Ivimey-Cook and Moorad (2018)]. Here we replicate this study design while modifying it in three ways. First, we expand the design from a biparental to a triparental ageing study by considering the joint influence of paternal age, which may reflect age-related differences in sperm quality or carcass preparation. Second, we survey more offspring traits, egg length and width, that are expected to correlate with fitness (Fox 1994; Xu et al. 2019). Finally, we experimentally increase larval density to generate a more stressful environment that we expect to intensify the main effects of parental age (ageing) and the pairwise interactions between parental ages (buffering against ageing).

## MATERIALS AND METHODS

### Study species

*Nicrophorus vespilloides* breed on carcasses of small vertebrates and display elaborate forms of parental care (Scott 1998). Upon acquiring a carcass, a mating pair prepare it for breeding by burying it, removing all fur, scales, or feathers, rolling the carrion into a ball, and treating it with antimicrobial secretions (Smiseth and Ward 2006). The female then lays eggs in the nearby soil. Newly hatched larvae aggregate on the carcass, where they both self-feed and are provisioned with pre-digested carrion by their parents, although the female is typically more involved with offspring care than the male. In this experiment, males were removed before larvae hatched and were thus prevented from providing post-natal care. Experimental removal of one parent does not appear to detrimentally affect offspring performance, and females are able to fully compensate for male absence (Smiseth et al. 2005). Parents care for their offspring until the larvae disperse from the carcass about 5 days after hatching.

The beetles used in this experiment were bred from a large, outbred stock population maintained at the University of Edinburgh. The stock population derived exclusively from a wild population sampled from Corstorphine Hill in Edinburgh, UK in 2016, and the experiment was performed in 2017. When not breeding, adults were housed individually in clear plastic containers (12 × 8 × 2 cm) filled with moist soil, at 20 °C, with a 16:8 light: dark photoperiod and were fed twice a week with raw organic beef.

### Experimental procedures

Female (egg-producer and foster mother) and male age at reproduction were classed as either “young” or “old” (11–18 days or 52–65 days post-eclosion). These age classes were chosen as they have differing levels of cumulative survival (94% and 26% respectively, Moorad personal communication), presumably leading to highly disparate intensities of selection for age-specific maternal effects that should favor the evolution of maternal senescence (Moorad and Nussey 2016). Sexual maturity is reached at around 10 days post-eclosion (Lock et al. 2007). Older ages were not used here due to the scarcity of beetles surviving beyond 65 days. Independently varying the age at reproduction for the egg-producer and the father yielded a full factorial 2 × 2 experimental design. We set up a total of 154 crosses.

**Figure 1:**
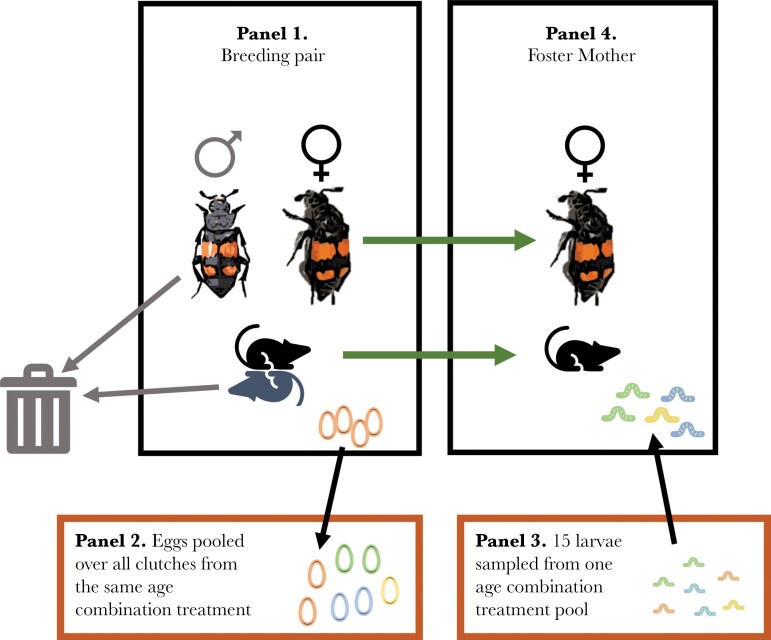
Experimental design. Three factors (parents) each have two levels corresponding to their ages (young/old). Panels 1–4 apply to any combination of these factors (e.g., an old father, a young egg-producer, and an old foster mother). Panel 1—the formation of breeding pair prior to disposal of the male and marked carcass; panel 2—pooling larvae over each age combination treatment; panel 3; sampling 15 larvae from each treatment to form a mixed brood; and panel 4—assigning a mixed brood to a foster mother who has earlier produced eggs. Note that the foster mothers are never given larvae from eggs that they produced. Image of adult male is adapted from a photo by Stanislav Snäll displayed at https://naturforskaren.se/species/f5a27e1b-f3db-45ce-b075-51fc334907c2.

We weighed each breeding pair before transferring them to a breeding box (17 × 12 × 6 cm) with 2 cm of moist soil containing two small mice carcasses (Figure 1; combined weight 17.59–25.63 g) (Livefood Direct Ltd, Sheffield, UK). For each mouse pair, we marked the carcass that deviated most from the average mouse weight of its experimental block by removing its tail. Females sense the size of the breeding resources (the carcass), and use that information to produce a number of eggs appropriate for that resource (Bartlett 1987; Trumbo 1990a). Removing the marked mouse from each pair allowed us to increase egg (and therefore larval) density beyond that which naturally occurs while also reducing the among-brood variation in remaining carcass size (and food resources) below that of the original pool of mice. In all cases, the beetles responded to the presence of two carcasses by rolling them together into a single ball.

### Egg size

142 of the 154 crosses produced eggs. We measured individual egg sizes by acquiring an image of the eggs laid 3 days after mating (when egg laying had ceased) through the bottom of the transparent breeding box using a Canon ConoScan 9000F Mark II (Canon Inc., Tokyo, Japan). Egg-laying usually begins 21 ± 2 h after a female is given access to a male and a carcass and lasts until 54 ± 3 h (Smiseth and Ward 2006). We assigned every visible egg in each image (mean = 8.49 ± 0.46 eggs) with a unique ID, and we measured each length and perpendicular width using ×600 magnification in the dedicated software *ImageJ 1.50i* (Schneider et al. 2012). After all eggs were measured once, we repeated the process to assess the repeatability of measurements. Overall, both the length and width of 1205 eggs were each measured twice (Supplementary Table S1); this resulted in a total of 4820 egg measurements. The correlations between first and second measurements of each egg were high (Spearman’s *ρ* = +0.90 for length and + 0.78 for width), indicating highly repeatable estimates.

### Cross-fostering

Females were weighed immediately after scanning eggs (3 days post-mating, see section above) to establish an initial weight for each female prior to providing care to offspring. Females were then transferred along with the unmarked carcass to new boxes containing only fresh soil (Figure 1). These were our pool of mothers from which we randomly drew foster mothers. We disposed of the marked carcasses and the males (as we did not assess post-natal paternal age effects; Figure 1). Each breeding box that contained eggs was checked regularly for hatched larvae [N.B. laying to hatching takes approximately 56 h at 20 °C (Smiseth and Ward 2006)]. Of the 142 crosses that produced eggs, 106 yielded larvae. We pooled larvae from egg-producer and paternal age groups to construct mixed broods of 15 larvae; each brood corresponded to one of the four treatment combinations (Figure 1). We chose a brood size of 15 because this represents a high but biologically reasonable number of larvae to be maintained on an 8.06–12.95 g carcass. This represents approximately twice the larval density (approximately 1.1 vs 0.6 larvae vs per gram of mouse) used in a previous *N. vespilloides* study of maternal senescence (Ivimey-Cook and Moorad 2018) and 50–100% greater density than was used in a *N. vespilloides* study of paternal senescence (Benowitz et al. 2013). We did this to increase the environmental stress placed upon offspring and foster mothers, as evidence suggests that more stressful conditions can exacerbate the deleterious effects of age upon survival (Lemaitre et al. 2013; Tidiere et al. 2016) and reproductive output (Lemaitre and Gaillard 2017; Cooper and Kruuk 2018). In this species, decreased resource availability (and thus increased environmental stress) has been found to exacerbate the deleterious effects of inbreeding (Richardson et al. 2018), and increased larval density is suggested to lead to more intense sibling competition and among-brood interference from individual larvae in obtaining resources (Smiseth et al. 2007).

Mixed broods from each of the four treatment combinations were distributed to young and old foster mothers (Figure 1). This resulted in a full factorial 2 × 2 × 2 experimental design (young/old for each of egg-producers, fathers, and foster mothers) with eight treatment combinations. As mixed broods were larger than the source broods; we had sufficient larvae to construct only 74 mixed broods. The numbers of mixed broods allocated to each treatment combination are given in Supplementary Table S2. Larval disperse and then pupate once the carcass is consumed. We counted and weighed each larva individually at dispersal for 73 mixed brood (one mixed brood yielded no survivors). The weight of larvae at dispersal is believed to indicate the degree of parental reproductive investment (Ward et al. 2009). Foster mothers were also weighed at dispersal, and these measures were compared to those taken immediately after egg laying. The weight change of foster mothers over the caring period is believed to be an indicator of female investment in this species (Creighton et al. 2009; Billman et al. 2014). We then transferred foster mothers to individual boxes where they were checked for death three times a week. This allowed us to correct for selective disappearance of foster mothers statistically by including foster mother age-of-death as a factor in our models (e.g., Ivimey-Cook and Moorad 2018).

### Statistical analyses

The experimental design required differently structured statistical models for different traits (summarized in Table 1). Univariate and bivariate linear mixed-models were analysed in ASReml 4.1 (Gilmour et al. 2015). The degree and nature of replication depended upon the specific traits. Egg length and width observations were made twice for each egg (2410 observations). Larval weight at dispersal was measured once for each offspring (697 observations). Foster mother weight change and larval survival rates were measured once per brood (73 observations). All main effects and two-way interactions between the egg-producer, paternal, and foster mother ages were included when biologically appropriate.

**Table 1:** Multivariate models used for data analysis

Trait	Model number	Parental age (fixed effects)^*^	Random effects ^†^	Replicates^‡^
Egg length Egg width	1	1.Egg-producer 2.Father 3.Egg-producer × father	1.Block 2.Foster mother ID 3.Egg ID	*n* *=* 2410 YY = 818 YO = 752 OY = 370 OO = 470
Larval weight at dispersal	2	1.Egg-producer 2.Father 3.Foster mother 4.Egg-producer × father 5.Egg-producer × foster mother 6.Father × foster mother	1.Block 2.Foster mother ID	*n* *=* 697 YYY = 114 YYO = 73 YOY = 88 YOO = 63 OYY = 83 OYO = 103 OOY = 90 OOO = 83
Foster mother weight change Larval survival to dispersal	3	1.Egg-producer 2.Father 3.Foster mother 4.Egg-producer × father 5.Egg-producer × foster mother 6.Father × foster mother	1.Block	*n* *=* 73 YYY = 12 YYO = 7 YOY = 10 YOO = 7 OYY = 8 OYO = 10 OOY = 9 OOO = 10

As carcass size is known to affect larval fitness and egg production (Müller et al. 1990; Trumbo 1990b), all models also included carcass weight and carcass weight^2^ as fixed effects. The combined weight of the two carcasses was used in the model to predict egg size, and the weight of the retained carcass was used for models predicting larval and foster mother outcomes.

ID effects were nested within block effects, and the effects of egg IDs were nested within the effects of egg-producer IDs to account for possible pseudo-replication.

Sample sizes are given as the total number in each analysis and as each individual age combination. Ages are in the order of egg-producer, father, and foster mother. For example, OYO indicates the number of old egg-producers × young fathers × old foster mothers.

Model 3 failed to converge initially and indicated a non-positive definite variance-covariance structure for block effects. In a similar manner to Ivimey-Cook and Moorad (2018), we ran univariate analyses for each female trait with and without the random effect of block to see if we could justify dropping this random effect from the full bivariate model. Likelihood ratio tests failed to find an effect of block upon the number of larvae surviving to dispersal (*P* = 0.906). However, there was a significant effect of block on foster mother weight change (*P* = 0.004). This justified rerunning the full model and only including the random effect of block for foster mother weight change (see Supplementary Table S3 for likelihood ratio test results).

Longitudinal age-related declines in reproductive performance can be masked by selective disappearance of poor-quality individuals from the population. This effect has been reported in studies of ageing in red deer (*Cervus elaphus*) (Nussey et al. 2011), Soay sheep (*Ovis aries*) (Hayward et al. 2013), and reindeer (*Rangifer tarandus*) (Weladji et al. 2008). However, this effect can be corrected for by including age of death as a covariate (van de Pol and Verhulst 2006; Nussey et al. 2011). Following Ivimey-Cook and Moorad (2018), we included foster mother age-at-death as a factor according to the interval during which death occurred. In this study, age had two levels: 1) between “young” and “old” age classes and 2) post “old” age class. The inclusion of foster mother age-at-death into statistical models allowed us to correct for the effects of selective disappearance of foster mothers, but not egg-producers or males, as the use of mixed broods precluded the identification of genetic parents.

We calculated *z-*scores for the parental age effect sizes by dividing them by standard errors, and *P* values were derived from these. Effect sizes were expressed as a daily rate by dividing by the median difference between the young and old age groups (44 days). This allows direct comparison with results from Ivimey-Cook and Moorad (2018).

## RESULTS

For brevity, only the model results for fixed age effects are provided here. Full model results are given as indicated below in the Supplemental section.

### Parental age effects on egg length and width

Old egg-producers and fathers produced eggs that were longer and narrower in comparison to young parents (Table 2). Interactions between egg-producer and father ages were negative but significantly less than zero only for effects on egg length. Differently put, matching the ages of the egg-producer and father was associated with decreased egg length.

**Table 2:** The effects of egg-producer and father age on egg size

Egg trait	Covariate	Effect size (10^‐3^mm/day)	Standard errors	*z-*score	*P*
Length	Egg-producer age	0.781	0.515	1.515	0.130
	Father age	0.179	0.445	0.402	0.688
	Egg-producer age × father age	‐1.551	0.690	‐2.246	**0.025**
Width	Egg-producer age	‐0.053	0.446	‐0.118	0.906
	Father age	‐0.044	0.392	‐0.113	0.910
	Egg-producer age × father age	‐0.580	0.596	‐0.974	0.330

The units of egg length and width are in mm. Effects that were significant to a threshold of *α* = 0.05 are in boldface. Full model results are given in Supplementary Table S4.

### Parental age effects on larval weight at dispersal

Larval weight decreased with increased age of the egg-producer, the father, and the foster mother. Both two-way interaction effects were positive: larval weight increased with when both parental ages increased, but neither interaction effect was statistically significant (Table 3, Supplementary Figure S2).

**Table 3:** The effects of egg-producer, father, and foster mother age on larval weight at dispersal

Covariate	Effect size (mg/day)	Standard errors (mg/day)	*z-*score	*P*
Egg-producer age	‐0.503	0.316	‐1.591	0.112
Father age	‐0.216	0.301	‐0.718	0.472
Foster mother age	‐0.270	0.323	‐0.834	0.404
Egg-producer age × father age	0.235	0.375	0.627	0.530
Egg-producer age × foster mother age	0.254	0.375	0.677	0.498
Father age × foster mother age	0.075	0.369	0.204	0.838
Foster mother age-at-death	‐0.814	0.800	‐1.017	0.309

Full model results are given in Supplementary Table S5.

### Parental age effects on foster mother traits, foster mother weight change, and larval survival to dispersal

Old foster mothers gained more weight than young foster mothers during the post-natal care period. However, both old fathers and old egg-producers produced larvae that caused foster mothers to gain less weight. All possible two-way interaction effects were positive, but none of the main or interaction effects were statistically significant. Larval survival increased with all three main effects of age, but only the main effect of egg-producer age was statistically significant. All two-way interactions were negative, but only the interaction of egg-producer and foster mother age was significant (Table 4, Supplementary Figures S3 and S4). Differently put, increased pre- and post-natal maternal age served to increase larval survival, but old age in one mother reduced the old-age advantage transmitted to the larvae from the other mother.

**Table 4:** Effects of egg-producer, father, and foster mother age on foster mother weight change and larval survival

Trait	Covariate	Effect size (unit/day)	Standard errors (unit/day)	*z*-score	*P* value
Foster mother weight change	Egg-producer age	‐0.160	0.344	‐0.466	0.641
	Father age	‐0.008	0.266	‐0.032	0.975
	Foster mother age	0.108	0.370	0.293	0.770
	Egg‐producer age × father age	0.195	0.383	0.509	0.611
	Egg-producer age × foster mother age	0.157	0.394	0.399	0.690
	Father age × foster mother age	0.143	0.345	0.414	0.679
	Foster mother age-at-death	‐0.332	0.738	‐0.450	0.653
Larval survival to dispersal	Egg-producer age	0.004	0.002	2.267	**0.023**
	Father age	0.001	0.002	0.713	0.476
	Foster mother age	0.003	0.002	1.807	0.071
	Egg-producer age × father age	‐0.002	0.002	‐0.951	0.342
	Egg-producer age × foster mother age	‐0.004	0.002	‐2.340	**0.019**
	Father age × foster mother age	‐0.003	0.002	‐1.658	0.097
	Foster mother age-at-death	0.004	0.004	0.958	0.338

Units are mg for weight change measurements, and larval survival is given in terms of a fraction (# survived divided by 15 for the initial brood size). Full model results are given in Supplementary Table S6.

## DISCUSSION

Past research to investigate bi-parental age effects has involved one of two experimental methodologies. Some studies use cross-fostering to disentangle the effects of pre- (egg-mediated) and post-natal (care-mediated) maternal age, most often in wild avian studies (Daunt et al. 2001; Beamonte-Barrientos et al. 2010; Schroeder et al. 2015), but cross-fostering has also been applied to investigate parental ageing in laboratory *N. vespilloides* populations (Lock et al. 2007; Ivimey-Cook and Moorad 2018). Other studies either exploit imperfect correlations between mother and father ages or they experimentally vary the ages of mothers and fathers separately to estimate the sex-specific contribution of each parent’s age (Bouwhuis et al. 2015; Fay et al. 2016; Tidiere et al. 2018). Here, we combine these two approaches to investigate the joint effects of the ages of three parents: 1) the egg producer, the female parent who provides genetic material and pre-natal care in the form of oviposition and carcass preparation; 2) the father, who in this experiment contributes only sperm and pre-natal care via carcass preparation; and 3) the foster mother, who cares for the larvae. Our analysis allowed us an unprecedented perspective on parental ageing by measuring it statistically with three main effects and three two-way age interactions. However, we found very little evidence for main effects of age, and this agrees with results found in a two-factor analysis of maternal ageing in the same study system (Ivimey-Cook and Moorad 2018). Our analysis more often revealed significant two-way interactions than significant main effects (one main effect of egg-producer age vs two interactions: egg-producer*father and egg-producer*foster mother). For these traits in this population, interactions appear to mitigate advantages to the offspring that are associated with older parents. More generally, these results suggest that among-parent interactions may be an important component of parental ageing, and these may suggest or reveal pathways that may not be identifiable from studies where the age of only one parent is considered. We discuss below how estimates from our study relate to those from other comparable studies and how our observations fit with what we understand about the life history of this *N. vespilloides*.

The size of eggs reflects the degree of maternal investment made into each egg (Bagenal 1969), and because this appears to predict the size of newly hatched *N. vespilloides* larvae (Monteith et al. 2012; Steiger 2013), egg size may be an important determinant of juvenile survival (and hence, fitness). If the resources available to a mother (or the ability of the mother to use these resources effectively) decline with age owing to senescence, then we might expect that egg size should also decline with maternal age. A previous study of hatch weight in this species found a negative effect of maternal age (Lock et al. 2007), but here we found no significant main effect of paternal and egg-producer age on egg length or width. In contrast, bird studies have reported maternal age-related *increases* in egg size (Weimerskirch 1992; Bogdanova et al. 2006). However, birds and insects may operate under very different constraints in terms of maternal investment in eggs, particularly in how they are subjected to size-number trade-offs that are expected to result from limited resources. In a smaller study (700 vs 1205 eggs), Monteith et al. (2012) reported no evidence of a negative correlation between egg size and clutch size in *N. vespilloides*, and they suggested that this may be because the costs of egg production are small in this species owing to their small size relative to the mothers. Although parental age was not directly manipulated in that study, they included maternal age as a covariate in their models and found that it had no significant relationship with egg size. Lastly, we note that increasing the size of the breeding resource had a significantly negative effect on egg length (*P *= 0.020, Supplementary Table S5). This could be in response to the perception by the female egg-producer that the environment would provide more resources for her larvae, which would therefore reduce the need to invest her own resources into her eggs. A pattern consistent with this has been observed in several Daphnia species (Guisande and Gliwicz 1992), where egg size increases with decreasing food abundance.

Survival to dispersal is of obvious importance to fitness in this species, as successful dispersal is a necessary condition for survival to adulthood. The evolutionary theory of senescence has thus far been applied to maternal effects formally for only those traits that are important components of neonatal survival (Moorad and Nussey 2016), and this theory predicts that juvenile survival should decline at old maternal ages. However, the current study found positive effects of both egg-producer and foster mother age on larval survival (0.004 and 0.003 survival/day respectively), although only the former was significantly different from zero. The direction and significance of both effects agrees with results from an earlier study from the same laboratory (0.02 and 0.01 survival/day respectively; published effect sizes were divided by 15 for comparison). However, it should be noted that model specifications differed (the older study contained quadratic effects of age) owing to a different experimental design (more than two ages were assessed in the previous study). We note that both studies detected a significant negative egg-producer-by-foster mother interaction effect on larval survival [‐0.004 and ‐0.0002 for estimates of here and by Ivimey-Cook and Moorad (2018)]. With the caveat that models are structured in slightly different ways, both results suggest that larval survival increases with the age of both the egg-producer and the foster mother, and the marginal increase in survival that a larva receives by having one old mother is suppressed if the other mother is old. It is not clear why this should be, but one possibility is that there are diminishing returns associated with benefits of ageing that are delivered to the offspring. Although we know of no study that has shown a similar effect of pre- and post-natal maternal ages on juvenile survival, interactions involving these two distinct ageing pathways are commonplace in cross-fostering experiments (Lock et al. 2007; Beamonte-Barrientos et al. 2010; Ivimey-Cook and Moorad 2018).

We found negative, but non-significant effects of the ages of foster mothers, egg-producers’, and fathers’ upon larval weight at dispersal. This trait predicts adult size, which is known to be an important factor in determining an adult’s competitive ability for securing reproductive resources (Bartlett 1988) and, therefore, for fitness. Ivimey-Cook and Moorad (2018) reported a significantly negative effect of egg-producer age but a positive (non-significant) effect of foster mother age. Ward et al. (2009) report significantly negative effects of increased maternal age on this trait, but their experimental design potentially conflated the effects of age with those of parity, and pre-natal and post-natal age effects could not be decoupled because that was not a cross-fostered experiment. The per-day effect of maternal age (combined over pre- and post-natal contributions) estimated in Ward et al. (2009) study was ‐1.97 mg/day [see Ivimey-Cook and Moorad (2018)]. We can arrive at a comparable estimate from the current study by summing the pre- and post-natal age effects. If we assume independence between the two, we can synthesize an SE associated with that measure by taking the square-root of the summed squares of the two components SEs. From this, we arrive at a total maternal age effect estimate of ‐0.77 mg/day with a SE of 0.80 mg/day. These imply a 95%-tile interval (‐2.025, 0.479) that includes the estimate from Ward et al. (2009). The same exercise applied to the relevant estimates taken from Ivimey-Cook and Moorad (2018) yields (‐0.951, 0.777), which includes the results presented here but not the results from Ward et al. (2009). We note, however, that the SEs estimated here are roughly half again as large as those reported by Ivimey-Cook and Moorad (2018), and this limits the power of this exercise to compare our results for combined maternal age effects with those from other studies. However, we can infer from our results that if these combined maternal age effects are deleterious with increased age, then they are likely no more extreme than those reported by Ward et al. (2009).

Lock et al. (2007) found no significant effect of the age of either the egg-producer or the foster mother on larval weight gain. As initial larval mass is approximately two orders of magnitude smaller than weight at dispersal (Lock et al. 2007), we can take weight gain to be a reasonable proxy for the latter, and these results can be viewed as consistent with ours. The earlier study found a positive interaction effect between the ages of egg-producers and foster mothers, which they interpreted as evidence for age-specific coadaptation for pre- and post-natal maternal effects. We also found a positive interaction, but it was non-significant. We note that this study used greater average brood sizes than Lock et al. (2007) (15 vs 9.3 larvae), suggesting that more resources were available to each individual offspring in the earlier study.

Burying beetles tend to increase their body mass by actively feeding from the carcass (Gray et al. 2018). Of course, this weight gain must be limited by the larvae feeding from the same resource. We found no statistical difference in the degree of weight gain between young and old foster mothers during post-natal care. Nor did the age of the egg-producer or father appear to influence the outcome of the foster mother. These results were similar to those reported by Ivimey-Cook and Moorad (2018). If the age of the genetic parents altered the burden placed upon the foster mother (by affecting the needs of the larvae, for example), then this burden was insufficient to affect foster mother investment enough to be detected. Further insights gained through behavioral observations could also help explain the lack of age effects on foster mother outcomes, particularly if parental care behaviors are changing because of increasing egg-producer and father age.

Given that female reproductive investment could be influenced by age-dependent characteristics of the partner, it is important to consider any paternal age-effects on reproductive performance. Studies that investigate these are rare and show mixed results, however. We found that paternal age has no detectable effect on offspring quality, aside from interacting with egg-producer age to alter egg length, in *N. vespilloides*; and this result is supported by Ward et al. (2009), who found no detectable effect upon mass at dispersal in this species. Fox et al. (2003) also found no evidence that paternal age affected any offspring traits in a study of the seed beetle *Callosobruchus maculatus*. However, Fay et al. (2016) found that the age of fathers in the wandering albatross, *Diomedea exulans,* significantly influenced offspring performance, with increasing paternal age leading to declines in juvenile survival. Paternal age in humans was shown to be the predominant factor determining the number of de novo mutations occurring within children (Kong et al. 2012), and increased paternal age in the houbara bustard, *Chlamydotis undulata*, was found to decrease sperm quality and hatching success and to slow neonatal development (Preston et al. 2015). Conversely, male age in a different bird species, *Colaptes auratus*, was associated with increased clutch size, earlier laying, and greater fledging success (Wiebe 2018). Results from the present study appear to support the notion that no clear patterns of directional effects exist across animal species.

The effects of differences between the age of father and mothers (parental age differences, or PADs) have received interest in studies of human reproduction, where they appear to predict family size (Fieder and Huber 2007; Helle et al. 2008). Tidiere et al. (2018) recently pointed out, however, that the effects of PADs are statistically entangled with the main effects of parental age, and for this reason they recommend that single parent ages be used as covariates in analyses of PAD effects. We agree with this reasoning, but Tidiere et al. (2018) leave out the statistically explicit definition of PADs provided here ($\beta_{z,PAD}=\beta_{z,M}-\beta_{z,F}-\beta_{z,M\times F}$), where main and interaction effects can be estimated simultaneously by GLMs (as we have done here). As we have noted, we detected effects of interactions between father and egg-producer upon egg length in *N. vespilloides.* In this case, effect sizes estimates given in Table 3 define the PAD effect to be equal to 0.949 × 10^‐3^ mm/day. Many other studies estimate parental-age-interaction effects (Laaksonen et al. 2002; Auld et al. 2013; Whelan et al. 2016), and these can be converted to define PAD effects in the same way to provide a clearer perspective of causality.

Selective disappearance has been shown to be an important contributor to population-level ageing patterns in wild vertebrate systems (Nussey et al. 2006; Hayward et al. 2013), but it is seldom investigated in laboratory studies of ageing. Our study controlled for selective disappearance of foster mothers, but we found no evidence that mortality biased parental age effects. Ivimey-Cook and Moorad (2018) also found no evidence for selective disappearance of foster mothers. Unfortunately, neither *N. vespilloides* study could investigate selective disappearance of egg-producers or father, as both experiments used mixed broods of larvae, and identification of the genetic parents of larvae was not possible. Using intact broods of cross-fostered larvae, where larval siblings were cross-fostered to a non-related foster mother, would have allowed us to account for the age of death of all three parents. However, a pilot study suggested that this would be extremely difficult owing to both asynchronous hatching of larvae within a brood and the high frequency of broods with small numbers of larvae. A further study using a larger combined carcass size would, in theory, increase the number of eggs laid and larvae produced by each breeding pair, and allow more successful cross-fostering of intact broods. In this way, potential bias arising from selective disappearance of the other parents could be assessed, too.

A recent review of the literature that examines maternal age effects on early survival (Ivimey-Cook and Moorad 2020) found that among widely-studied taxa, insect species demonstrate the strongest tendencies towards maternal senescence (17 of 26 species exhibited senescence). As our results show that maternal senescence is not manifested on several important offspring traits; this suggests that *N. vespilloides* is unusual. This species has a peculiar life history that may indicate an evolutionary cause for slowed (and, thus, harder to detect) rates of maternal effect senescence. Evolutionary theory argues that selection for age-specific maternal effects upon neonatal survival is proportional to the age distribution of maternal ages (Moorad and Nussey 2016). It must follow that when reproduction is highly focused upon a small range of ages, then the decline in selection for maternal effects that follows the age that maximizes this distribution (the mode) will be dramatic, and evolution will favor extreme rates of ageing. When reproduction is spread over many ages, the opposite is expected: ageing should be gradual. Reproduction in *N. vespilloides* depends upon the availability of vertebrate carcasses, and this is a rare and unpredictable resource (Scott 1998). We should expect high variation in the age of first reproduction and in the time between successive reproductive events as a result. These features will cause the shape of the frequency distribution of maternal ages at birth to favor a later mode (the age of strongest selection) and subsequent slow declines afterwards (a slow rate of relaxing selection). The latter feature will promote strong selection to resist senescence in maternal age effects, which is consistent with the observations made here for larval survival. Whilst the evolutionary theory makes explicit predictions regarding only maternal effects on neonatal survival, it seems reasonable to expect that similar predictions would apply both to other offspring traits and to paternal age effects, but future models should be developed to generalize the theory to consider these other traits and factors.

## Supplementary Material

arac078_suppl_Supplementary_Material

## Notes

The authors would like to thank Per Smiseth and members of the Moorad and Smiseth lab groups for laboratory assistance and helpful commentary during the preparation of this manuscript. HC was supported by an Institute of Evolutionary Biology Summer Studentship, and ERIC was supported by an East of Scotland Bioscience Doctoral Training Partnership funded by the Biotechnology and Biological Sciences Research Council (544EIC BB/J10446X/1). We would also like to thank the editors and two anonymous reviewers for their helpful and insightful feedback during the review process.

Analyses reported in this article can be reproduced using the data provided by Cope et al. (2022).
