# Supplementary material for: Triparental ageing in a laboratory population of an insect with maternal care
Source: Behav Ecol. 2022 Aug 24;33(6):1123–32. doi: 10.1093/beheco/arac078 (PMC9735237; doi:10.1093/beheco/arac078)
Supplement: arac078_suppl_Supplementary_Material [file arac078_suppl_supplementary_material.docx]

**Supporting Information for:**

“Triparental aging in a laboratory population of an insect with maternal care*”*

Hilary Cope, Edward Ivimey-Cook and Jacob Moorad*

**Table S1.** Eggs measured in each treatment

| Treatment (Egg-producer age, father age) | Number of eggs measured |
| --- | --- |
| Young, Young | 409 |
| Young, Old | 376 |
| Old, Young | 185 |
| Old, Old | 235 |
| Total | 1205 |

**Table S2.** Number of cross-fostered broods achieved, and the total number of surviving larvae at dispersal in each treatment

| Treatment (Egg-producer age, father age, foster mother age) | Number of broods | Number of larvae |
| --- | --- | --- |
| Young, Young, Young | 12 | 114 |
| Young, Young, Old | 7 | 73 |
| Young, Old, Young | 10 | 88 |
| Young, Old, Old | 7 | 63 |
| Old, Young, Young | 8 | 83 |
| Old, Young, Old | 10 | 103 |
| Old, Old, Young | 9 | 90 |
| Old, Old, Old | 10 | 83 |
| Total | 73 | 697 |

**Table S3**. Likelihood ratio tests for bivariate foster mother-level models with and without block effects

| Trait | Log-likelihood with block | Log-likelihood without block | *D* | p value |
| --- | --- | --- | --- | --- |
| Foster mother  weight change | 188.293 | 184.108 | 8.370 | **0.00381** |
| Larval survival to dispersal | 108.090 | 108.097 | 0.014 | 0.906 |

**Table S4.** Parameter estimates from the full bivariate model assessed at the level of the egg.

| Trait | Covariate | Effect size  (10^-3^ mm/day) | Standard errors | z score | p -value |
| --- | --- | --- | --- | --- | --- |
| Egg length | **Intercept** | **56.522** | **0.306** | **184.633** | **<0.001** |
|  | Egg-producer age | 0.781 | 0.515 | 1.515 | 0.130 |
|  | Father age | 0.179 | 0.445 | 0.402 | 0.688 |
|  | **Egg-producer age*father age** | **-1.551** | **0.69** | **-2.246** | **0.0247** |
|  | **Carcass weight** | **-0.771** | **0.333** | **-2.320** | **0.0203** |
|  | Carcass weight^2^ | -0.622 | 0.645 | -0.964 | 0.335 |
| Egg width | **Intercept** | **29.614** | **0.271** | **109.404** | **<0.001** |
|  | Egg-producer age | -0.0528 | 0.446 | -0.118 | 0.906 |
|  | Father age | -0.0442 | 0.392 | -0.113 | 0.910 |
|  | Egg-producer age*father age | -0.580 | 0.596 | -0.974 | 0.330 |
|  | Carcass weight | 0.0441 | 0.284 | 0.155 | 0.877 |
|  | Carcass weight^2^ | -0.456 | 0.553 | -0.825 | 0.409 |

**Table S5.** Parameter estimates from the full univariate linear model assessed at the level of the larvae

| Trait | Covariate | Effect size  (mg/day) | Standard errors | z score | p |
| --- | --- | --- | --- | --- | --- |
| Larval weight at dispersal | **Intercept** | **5.127** | **0.220** | **23.279** | **<0.001** |
|  | Egg-producer age | -0.503 | 0.316 | -1.591 | 0.112 |
|  | Father age | -0.216 | 0.301 | -0.718 | 0.472 |
|  | Foster mother age | -0.270 | 0.323 | -0.834 | 0.404 |
|  | Egg-producer age*father age | 0.235 | 0.375 | 0.627 | 0.530 |
|  | Egg-producer age*foster mother age | 0.254 | 0.375 | 0.677 | 0.498 |
|  | Father age*foster mother age | 0.0752 | 0.369 | 0.204 | 0.838 |
|  | Age of foster mother at death | -0.814 | 0.800 | -1.017 | 0.309 |
|  | **Carcass weight** | **0.585** | **0.236** | **2.476** | **0.0133** |
|  | Carcass weight^2^ | 0.383 | 0.347 | 1.103 | 0.270 |

**Table S6.** Parameter estimates from the full bivariate model assessed at the level of the foster mother.

| Trait | Covariate | Effect size  (unit/day) | Standard errors | z score | p |
| --- | --- | --- | --- | --- | --- |
| Foster mother weight change | Intercept | -0.530 | 0.325 | -1.633 | 0.102 |
|  | Egg-producer age | -0.160 | 0.344 | -0.466 | 0.641 |
|  | Father age | -0.00843 | 0.266 | -0.0316 | 0.975 |
|  | Foster mother age | 0.108 | 0.370 | 0.293 | 0.770 |
|  | Egg-producer age*father age | 0.195 | 0.383 | 0.509 | 0.611 |
|  | Egg-producer age*foster mother age | 0.157 | 0.394 | 0.399 | 0.690 |
|  | Father age*foster mother age | 0.143 | 0.345 | 0.414 | 0.679 |
|  | Age of foster mother at death | -0.332 | 0.738 | -0.450 | 0.653 |
|  | Carcass weight | -0.198 | 0.226 | -0.874 | 0.382 |
|  | Carcass weight^2^ | 0.475 | 0.326 | 1.459 | 0.145 |
| **Larval survival to dispersal** | **Intercept** | **0.0130** | **0.00110** | **11.797** | **<0.001** |
|  | **Egg-producer age** | **0.00363** | **0.00160** | **2.267** | **0.0234** |
|  | Father age | 0.00110 | 0.00155 | 0.713 | 0.476 |
|  | Foster mother age | 0.00296 | 0.00164 | 1.807 | 0.0708 |
|  | Egg-producer age*father age | -0.00181 | 0.00190 | -0.951 | 0.342 |
|  | **Egg-producer age*foster mother age** | **-0.00447** | **0.00191** | **-2.340** | **0.0193** |
|  | Father age*foster mother age | -0.00313 | 0.00189 | -1.658 | 0.0973 |
|  | Age of foster mother at death | 0.00397 | 0.00414 | 0.958 | 0.338 |
|  | **Carcass weight** | **-0.00287** | **0.00120** | **-2.401** | **0.0164** |
|  | Carcass weight^2^ | -0.00197 | 0.00176 | -1.119 | 0.263 |

**
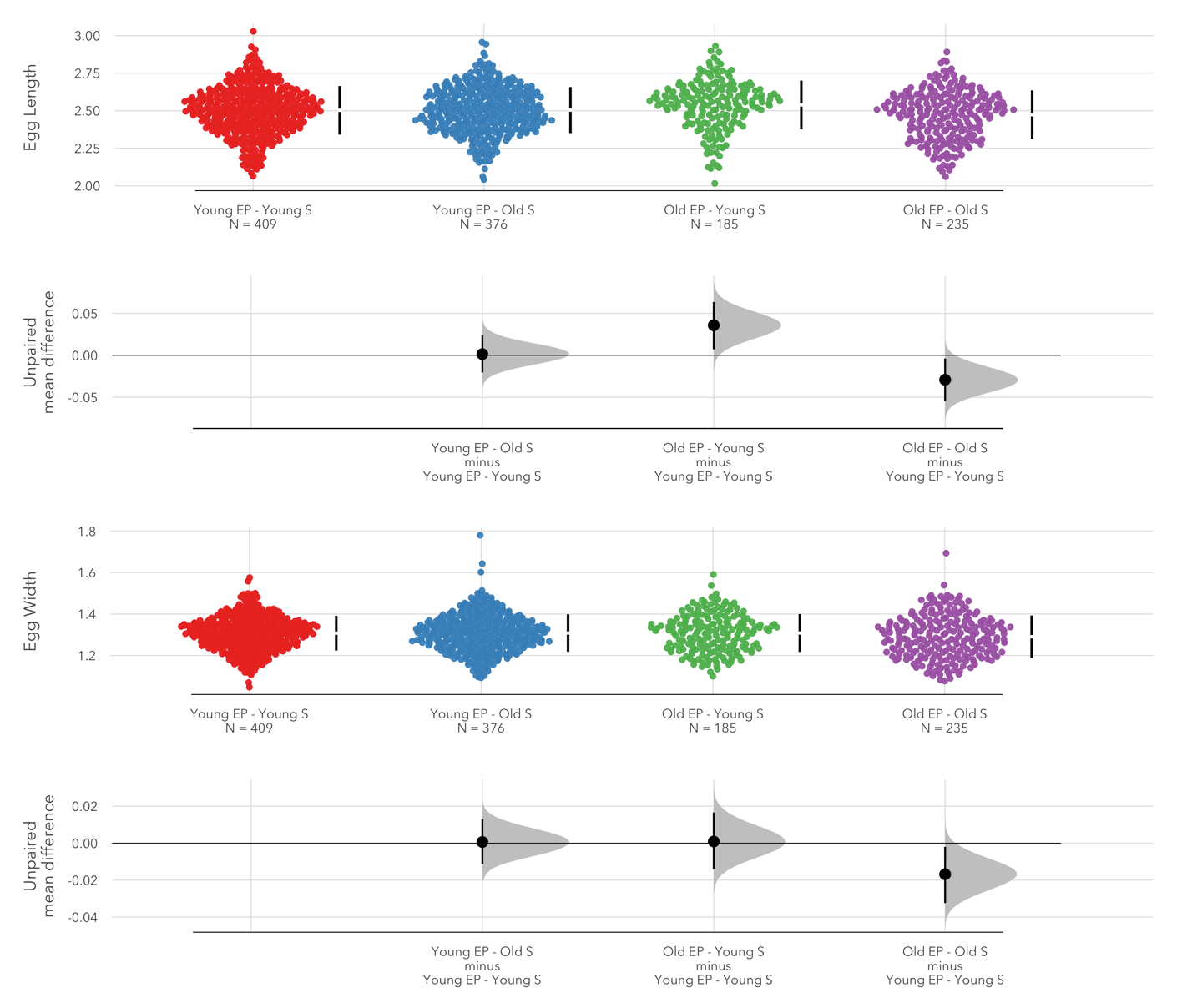
**

**Figure S1.** Effects of egg-producer (EP) and sire (S) age on egg length (top) and width (bottom). The top panel shows the distribution of egg measurements with mean and bootstrapped 95% confidence intervals. The bottom plot shows the bootstrapped mean difference between the reference level (Young EP -Young S) and other combinations of egg-producer and sire ages.


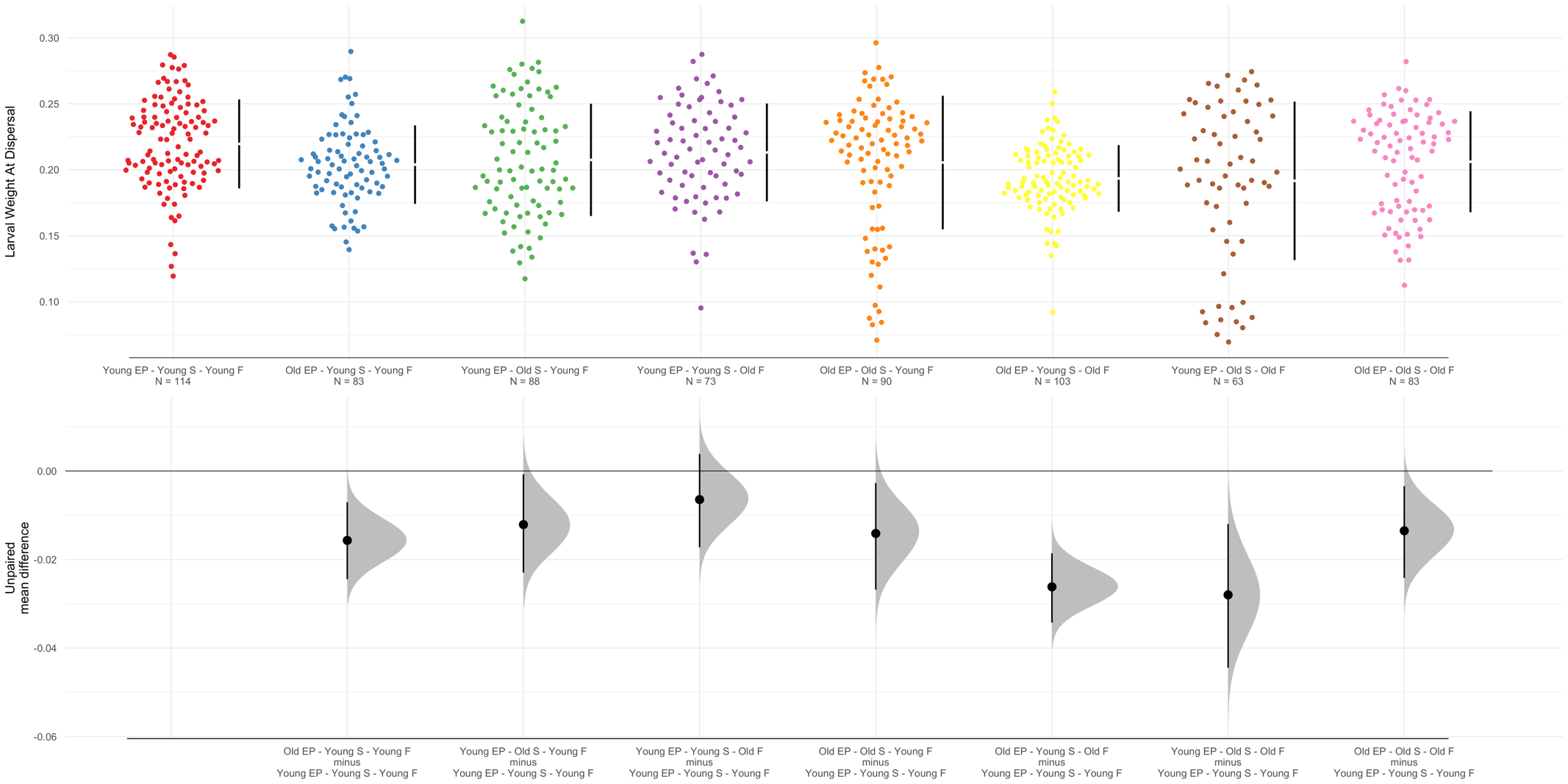


**Figure S2.** Effect of egg-producer (EP), sire (S) and foster mother (F) age on larval weight at dispersal. The top panel shows the distribution of larval dispersal weights with mean and bootstrapped 95% confidence intervals. The bottom plot shows the bootstrapped mean difference between the reference level (Young EP - Young S - Young F) and other combinations of egg-producer, sire, and foster mother ages.

**
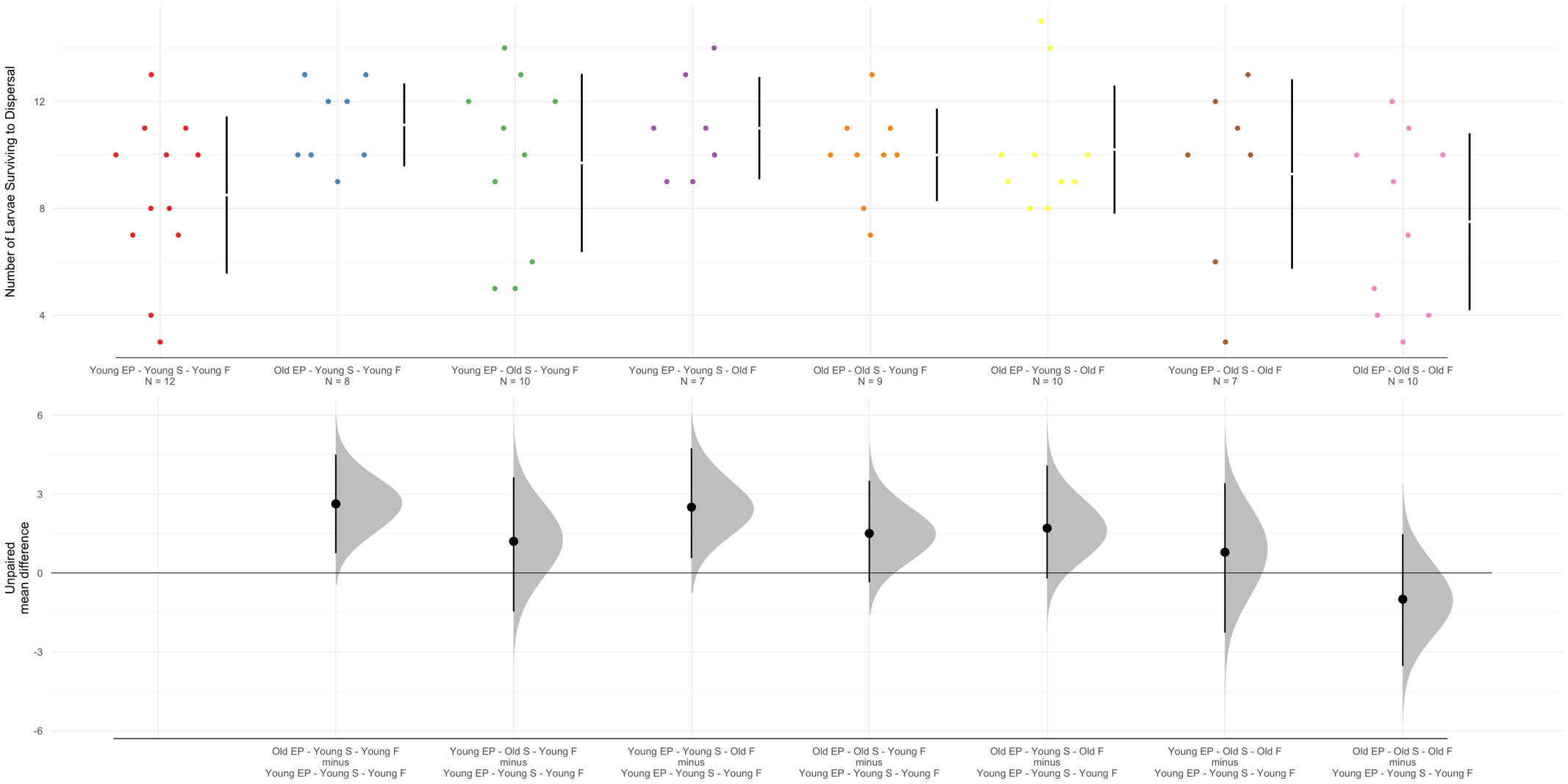
**

**Figure S3** Effect of egg-producer (EP), sire (S) and foster mother (F) age on number of larvae surviving to dispersal. The top panel shows the distribution of surviving larval number for each female with mean and bootstrapped 95% confidence intervals. The bottom plot shows the bootstrapped mean difference between the reference level (Young EP -Young S - Young F) and other combinations of egg-producer, sire, and foster mother ages.


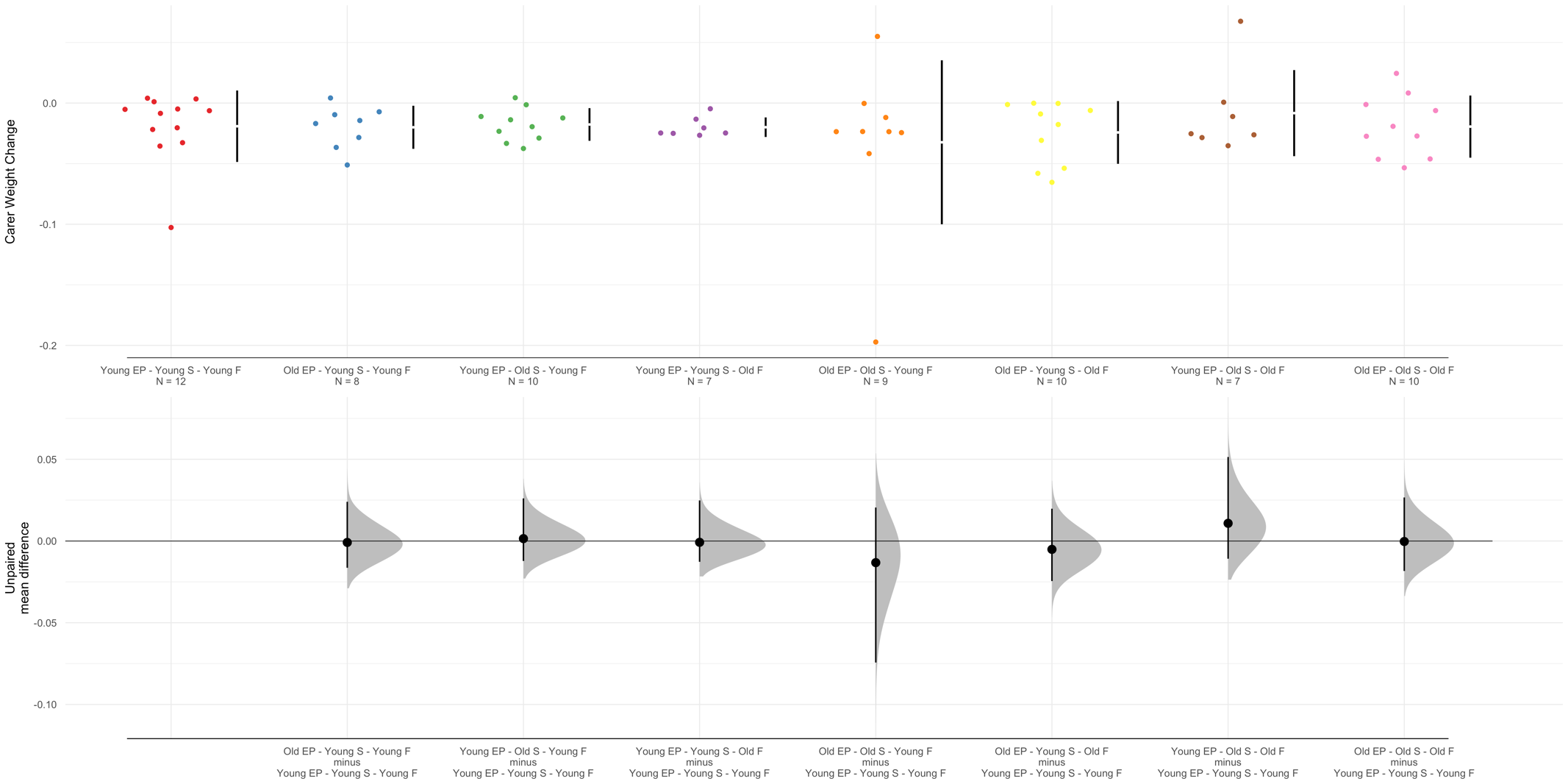


**Figure S4.** Effect of egg-producer (EP), sire (S) and foster mother (F) age on foster mother weight change. The top panel shows the distribution of foster mother weight change for each female with mean and bootstrapped 95% confidence intervals. The bottom plot shows the bootstrapped mean difference between the reference level (Young EP - Young S - Young F) and other combinations of egg-producer, sire, and foster mother ages.
